# Identification of Natural Compounds Triggering MRGPRX2-Mediated Calcium Flux and Degranulation in RBL-2H3 Cells

**DOI:** 10.3390/cells15030287

**Published:** 2026-02-03

**Authors:** Lihui Zhang, Jing Liu, Jian Zheng, Wenguang Jing, Wenjuan Zhang, Jia Chen, Xinyue Zhang, Xianlong Cheng, Feng Wei

**Affiliations:** 1 Institute for Control of Traditional Chinese Medicine and Ethnic Medicine, National Institutes for Food and Drug Control, Beijing 102629, China; 2 State Key Laboratory of Drug Regulatory Science, National Institutes for Food and Drug Control, Beijing 102629, China

**Keywords:** anaphylactoid reactions, MRGPRX2, natural compounds, mast cells (MCs), tryptase, β-hexosaminidase

## Abstract

**Highlights:**

**What are the main findings?**

- Established a human MRGPRX2-overexpressing cell model that exhibits enhanced sensitivity to mast cell degranulation.
- Computer-assisted screening identified natural compounds capable of inducing mast cell degranulation and triggering anaphylactoid reactions.

**What are the implications of the main findings?**

- The established cell model is suitable for in vitro research on anaphylactoid reactions.
- This study offers valuable insights for drug safety warnings, dosage control, and mechanistic discovery.

**Abstract:**

Natural compounds have experienced increasing clinical application, but their association with rapid-onset anaphylactoid reactions (ARs) present a significant challenge to their safe use. These ARs, clinically resembling Type I hypersensitivity, are non-IgE-mediated and involve direct mast cell activation, primarily through the human Mas-related G protein-coupled receptor X2 (MRGPRX2). We computationally screened a natural compound library for MRGPRX2 activation. A human MRGPRX2-expressing cell model was established. Cell viability assays (0–80 μM) were performed to determine appropriate drug concentrations. Compared to the controls, Baohuoside I (10 μM), along with Kaempferol-3-O-rutinoside, Epigallocatechin gallate (EGCG), Isochlorogenic Acid B, Baicalin, Andrographolide, Isorhamnetin, and Dehydroandrographolide (all at 20 μM), significantly increased intracellular calcium flux (*p* < 0.05) and boosted tryptase and β-hexosaminidase secretion (ELISA) (*p* < 0.05) in mast cells. Furthermore, the degranulation induced by these compounds was inhibited by the MRGPRX2 inhibitor Z3578 at 20 μM. Neutral red staining was employed to observe cellular morphological changes. Specific compounds capable of mediating ARs through MRGPRX2 activation on mast cells were identified. This contributes to safer and more effective drug use by elucidating the potential triggers of ARs.

## 1. Introduction

The expanding application of natural compounds has brought increasing attention to its associated adverse reactions. Specifically, rapid-onset anaphylactoid reactions (ARs) induced by Traditional Chinese Medicine Injections (TCMIs) represent a significant hurdle to their safe and effective clinical use. Data from the National Center for Adverse Drug Reaction Monitoring reveal that between 2011 and 2018, TCMI adverse drug reactions/events (ADR/ADE) comprised over half of all Traditional Chinese Medicine (TCM)-related ADRs in China, notably surpassing those from oral or other administration routes [1]. However, a subsequent positive trend was observed from 2019 to 2023, with a decline in TCMI ADR/ADE incidence, largely attributed to ongoing post-marketing safety re-evaluation efforts and enhanced clinician awareness regarding rational drug use [2,3].

Overall, ARs constitute the predominant type of adverse reactions/events associated with TCMI, and their proportion is relatively high among severe adverse event reports. For instance, among the 33 TCMIs listed in the China National Essential Drug List (2004 edition), all have reported clinical adverse events, with ARs accounting for 75% of these events [4]. TCMI-induced ADRs characteristically present as acute allergic reactions, frequently manifesting within 30 min of initial administration [5]. Approximately 77% of acute clinical hypersensitivity reactions are identified as ARs [6]. Although their clinical symptoms resemble those of Type I hypersensitivity reactions, their underlying mechanisms are fundamentally distinct [7].

Anaphylactoid reactions are acute systemic reactions that are non-IgE-mediated and clinically resemble allergic reactions. These reactions involve the direct activation of mast cells/basophils [8,9], leading to the degranulation of target cells and the release of allergic mediators [10]. In 2006, it was first identified that ARs are mediated by human MRGPRX2 present on the surface of mast cells [11]. Subsequently, Mrgpr X2 and Mrgprb2 were found to be targets for many small molecule drugs associated with systemic pseudoallergic or ARs [12,13]. In drug-induced non-immune hypersensitivity reactions, nanoparticles and biologics primarily induce anaphylactoid reactions by activating the complement system, which may be related to macromolecules involved in complement system activation pathways [14]. In contrast, certain small molecule compounds within TCMIs may rapidly trigger mast cell degranulation upon binding with MRGPRX2 [15], leading to the release of abundant inflammatory mediators such as histamine. This pathway operates independently of IgE and the complement system, explaining why some natural compounds can induce rapid adverse reactions without prior sensitization.

The intricate chemical composition of TCM includes numerous structurally diverse small molecules such as alkaloids, flavonoids, saponins, and terpenes. Notably, some components have been reported to influence allergic/anaphylactoid reactions. For instance, severe anaphylactic shock observed clinically following the administration of Houttuynia Cordata injection suggests that houttuyfonate might be a key allergenic component [16]. In Honeysuckle, chlorogenic acid and cryptochlorogenic acid have been demonstrated to elevate plasma serotonin and β-hexosaminidase levels in guinea pigs and induce degranulation of RBL-2H3 cells, thereby triggering anaphylactoid reactions [17]. Furthermore, an ethanol extract of 80% Red Ginseng has been shown to induce degranulation of RBL-2H3 and LAD2 cells [18]. Andrographis paniculata extracts led to increased weight and cellularity indices of popliteal lymph nodes in BALB/c mice, and markedly elevated the level of histamine, the percentage of cell degranulation, and the ratio of ammonia glycosidase released from P815 cells [19]. Conversely, studies have indicated that kaempferol can ameliorate IgE-FcεRI-mediated anaphylaxis in C57BL/6 mice, bone marrow-derived mast cells, and LAD2 cells [20,21]. Genistein, on the other hand, has been found to inhibit MRGPRX2/MrgprB2-mediated anaphylactoid reactions activated by C48/80 in LAD2 cells and mouse models [22]. Among these, certain compounds, particularly those with a positive charge or amphiphilic structure, are considered potential ligands for MRGPRX2. However, systematic research and direct evidence regarding which specific single natural compounds of TCM can directly activate MRGPRX2 are currently lacking.

In this study, we first employed computational molecular docking and simulation techniques, targeting the MRGPRX2 receptor structure, to conduct a large-scale virtual screening of the marker natural compounds library listed in the Chinese Pharmacopoeia. Using potent agonists as references, we preliminarily predicted small molecules with potential binding affinity to the receptor’s activation site. Subsequently, we determined appropriate drug concentrations using cell viability assays in MRGPRX2-expressing cells, and observed cell morphology via neutral red staining. Finally, the ability of these natural compounds to functionally induce mast cell degranulation was verified by measuring degranulation indicators.

## 2. Materials and Methods

### 2.1. Chemicals

Compound 48/80 (C48/80) (HY-115768), (R)-ZINC-3573 (HY-118069), substance P (HY-P0201), PAMP-12 (HY-P3419), Z-3578 (HY-172458), Mogroside V (HY-N0502), Cornuside (HY-N0631), Baohuoside I (HY-N0011), Kaempferol-3-O-rutinoside (HY-N8165), EGCG (HY-13653), Quercetin-3-O-Glucuronide (HY-13930), Isochlorogenic Acid B (HY-N0057), oroxylin A 7-O-β-D-Glucuronide (HY-N2481), 2,3,5,4-tetrahydroxyl diphenylethylene-2-o-glucoside (THSG) (HY-N0652), and Prunasin (HY-N1548) were purchased from MedChem Express (Monmouth Junction, NJ, USA). Rutin (100080-202513), Baicalin (112002-202303), Vitexin (111687-202306), Andrographolide (110797-202010), Isorhamnetin (110860-202513), Arbutin (111951-201301), Dehydroandrographolide (110854-202412), Crotonoside (111856-201102), Chrysin (111701-202102), Baicalein (111595-202309), and Cordycepin (110858-201704) were obtained from National Institutes for Food and Drug Control (Beijing, China). MEM (Lot No. 11095080), PBS buffer (Lot No. 1897215), trypsin (Lot No. 1951055), and fetal bovine serum (Lot No. A2892802RP) were all purchased from Gibco (Grand Island, NY, USA). The purity of all compounds in this study was above 98%.

### 2.2. Molecular Docking

The crystal structure of human MRGPRX2 (PDB ID: 7VV4) was retrieved from the Research Collaboratory for Structural Bioinformatics Protein Data Bank (RCSB PDB). The binding site for small molecule agonists, namely C48/80, (R)-ZINC-3573, PAMP-12, and Substance P, served as a reference for defining the active site. The following pocket amino acid residues were identified and considered for docking: ARG220, THR224, ARG127, TRP243, THR106, PHE239, THR110, TYR113, GLY236, PHE170, GLU164, ASP184, CYS180, CYS168, PHE257, SER177, SER253, ASP254, LEU247, TRP248, PHE239, TYR113, THR187, SER268, ASN271, GLY236, GLY116, PHE232, LEU191, ILE192, PRO262, TRP179, ASN85, VAL88, CYS95, TYR89, THR106, PHE239, TYR113, THR187, SER268, ASN271, GLY236, GLY116, PHE232, LEU191, ILE192, CYS258, PRO262, TRP179, ASN85, VAL88, CYS95, TYR89, THR106, HIS261, MET109, ILE84, PHE105, PRO102, and ASP176. A library of 496 marker natural compounds, representative of the diverse structures and bioactivities found in TCM, was compiled based on the Chinese Pharmacopoeia. Prior to docking, structural preparation of both the target protein and ligand molecules was performed using the QuickPrep module within the Molecular Operating Environment (MOE) software (version 2019.01).

In this study, a binding energy (S) less than −6 kcal·mol^−1^ was set as the primary critical threshold for initial consideration. Furthermore, compounds exhibiting a docking score less than −5 kcal·mol^−1^ and forming at least five interaction bonds with the protein were considered to demonstrate significant binding efficacy.

### 2.3. Cell Culture

Rat basophilic leukemia (RBL-2H3) cells (CL-0192) were purchased from Procell Life Science & Technology Co., Ltd., Wuhan, China. A stable H_MRGX2 RBL-2H3 cell line overexpressing human MRGPRX2 was generated in our laboratory. Hereafter, the term ‘H_MRGX2 RBL-2H3’ will be used to refer to these cells. H_MRGX2 cells were cultured in MEM supplemented with 10% fetal bovine serum (FBS), 100 U/mL penicillin, and 100 U/mL streptomycin, and maintained at 37 °C in a humidified atmosphere containing 5% CO_2_. Cells in the logarithmic growth phase were used for all experiments. HMC1.2 cells (Thermo Fisher Scientific, Waltham, MA, USA, #A3840101) were cultured in IMDM (GIBCO, Grand Island, NY, USA, 12440053) supplemented with 10% inactivated fetal calf serum (FCS) and 1% antibiotics (penicillin/streptomycin, Lonza, Verviers, Belgium). This cell line has previously been shown to express the MRGPRX2 receptor [15]. Hereafter, the term ‘HMC-1’ will be used to refer to these cells.

### 2.4. Compound Intervention

The experiment included a control group, positive control drug groups, and natural compounds intervention groups. Based on their solubility, drugs were completely dissolved in DMSO to prepare stock solutions. For experiments, these stock solutions were diluted to the desired concentrations (20, 40, 80 µM) using serum-free MEM and IMDM. Cells were treated with the respective drug solutions for 1 h before subsequent measurement of relevant indicators.

### 2.5. H_MRGX2 Antibody Staining

RBL-2H3 cells and H_MRGX2 RBL-2H3 cells were seeded at a density of 8 × 10^5^ cells per well in a six-well plate. After 24 h, 1 μg/mL of PE anti-human MRGX2 Antibody (BioLegend Co., Ltd., San Diego, CA, USA, Cat.#359002) working solution was added. The cells were then incubated at 37 °C and 5% CO_2_ for 30 min, followed by three washes with serum-free cell culture medium. Detection was performed using a laser confocal microscope and flow cytometry.

### 2.6. Cell Viability Assay

H_MRGX2 RBL-2H3 cells in the logarithmic growth phase were seeded into 96-well plates at a density of 5000 cells/well. After 24 h, the culture medium was replaced with serum-free MEM containing the test compounds at various concentrations (0, 20, 40, and 80 μM). Each treatment group included 4–6 replicates. Following a 1 h incubation at 37 °C in a 5% CO_2_ atmosphere, 10 μL of CCK-8 solution was added to each well, and the plates were incubated in the dark for 1 h. Absorbance was measured at 450 nm using a microplate reader. Cell viability was calculated as follows:

Cell Viability (%) = [(Absorbance of Treated Group − Absorbance of Blank Group)/(Absorbance of Control Group − Absorbance of Blank Group)] × 100%

### 2.7. Flow Cytometry

Cells were seeded into 12-well plates at a density of 1 × 10^6^ cells/well and treated with drugs for 1 h according to their respective groups. A stock solution of the Fluo-4 AM (Thermo Fisher Scientific, Waltham, MA, USA, F14201) calcium indicator was diluted with PBS to prepare a working solution with a concentration of 0.5–5 µmol/L. Cells in each group were then incubated with the Fluo-4 AM working solution at 37 °C for 30 min to allow for fluorescent probe loading. Following incubation, cells were washed three times with PBS. Finally, 400 µL of 1× binding buffer was added to each sample, and intracellular calcium levels were analyzed using a flow cytometer.

### 2.8. ELISA

Cell culture supernatants collected after group-specific interventions were analyzed using an ELISA kit according to the manufacturer’s instructions. Each sample was tested in triplicate alongside standard wells. Briefly, 100 μL of standards or samples were added to the respective wells and incubated at 37 °C for 2 h. Subsequently, 100 μL of biotin-labeled detection antibody working solution was added to each well and incubated at 37 °C for 1 h. After incubation, 100 μL of HRP-conjugated streptavidin was added to each well and incubated at 37 °C for an additional hour. The substrate solution (90 μL) was then added to each well, and color development was performed at 37 °C in the dark for 30 min. The reaction was terminated by adding 50 μL of stop solution to each well. Absorbance at 450 nm was measured within 5 min using a microplate reader. The concentration of analytes was determined by interpolating from the standard curve.

### 2.9. Neutral Red Staining

H_MRGX2 RBL-2H3 cells were seeded into 24-well plates at a density of 5 × 10^4^ cells/well. After 24 h of incubation, the culture medium was aspirated, and cells were washed once with PBS. Subsequently, drugs diluted in serum-free MEM were added to the respective wells. Serum-free MEM served as a negative control. Each treatment group included three replicate wells. Following 1 h of incubation in a cell culture incubator, the supernatants were aspirated. Cells were then stained by adding 300 µL of 0.5% neutral red solution to each well for 5 min. After aspirating the neutral red solution, representative images were captured using an optical microscope at 100× magnification.

### 2.10. Statistical Analysis

Differences between two groups were assessed using the Student’s *t*-test. For comparisons of means among multiple groups, a one-way analysis of variance (ANOVA) was employed. Post hoc comparisons were conducted using the Least Significant Difference (LSD) test when homogeneity of variance was confirmed. In cases of unequal variances, Dunnett’s T3 test was used for pairwise comparisons. Statistical significance was considered at *p* < 0.05. Graphical results were generated using GraphPad Prism 9 software.

## 3. Results

### 3.1. A Top-Ranked Natural Compounds Selected Based on the MRGXPRX2 Protein Binding Affinity and Docking Performance

PubChem CID represents the compound’s unique identifier in the PubChem database. Name is the compound’s name. Mol denotes the molecular structure. S stands for the final docking score (S); it is a numerical value used by the docking program to evaluate the binding affinity between the ligand and the receptor. Lower scores indicate higher affinity and more stable binding. rmsd_refine refers to the root-mean-square deviation between the ligand’s conformation after refinement and the reference conformation. E_conf is the ligand’s conformational energy, reflecting the energy of the ligand’s own conformation and its stability in that conformation. E_place typically refers to the energy during the placement step (Table 1). E_score is the docking scoring energy, reflecting the strength of the interaction between the ligand and receptor. E_refine is the energy or score during the refinement process; after placing the ligand, the docking program optimizes its position and conformation to achieve a more stable binding mode.

The subsequent experiments will use these natural compounds, but because Aconitine is a controlled substance with high toxicity and there are safety and supply constraints, Aconitine was not used in the follow-up experiments. Instead, DMSO was used as a solvent control group.

### 3.2. Validation of RBL-2H3 Cell Line with High Expression of H_MRGX2

**Figure 1:**
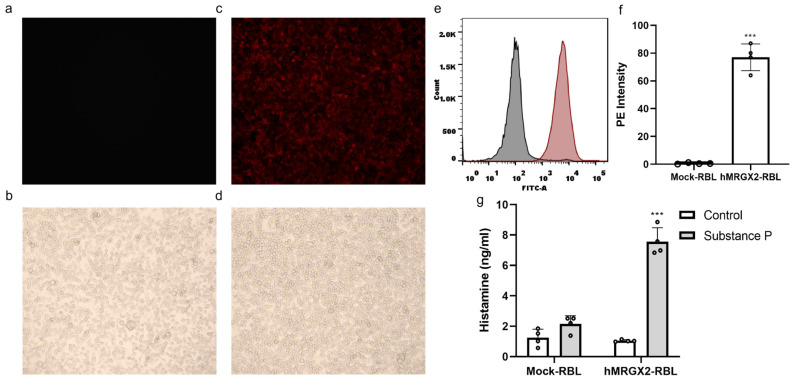
Fluorescent antibody staining of RBL-2H3 cells and MRGPRX2-overexpressing RBL-2H3 cell receptors (**a**,**c**), observation of RBL-2H3 cells and MRGPRX2-overexpressing RBL-2H3 cells under a bright-field microscope (100×) (**b**,**d**). Flow cytometry fitting curves of Mock-RBL-2H3 and H_MRGX2-RBL groups (**e**) and statistical results of average fluorescence intensity (**f**) (*n* = 4 per group). *** *p* < 0.001 versus Mock-RBL. (**g**) Effect of substance P on histamine content in two cell types (*n* = 4 per group). *** *p* < 0.001 versus Control; Student’s *t*-test; mean (SD).

We performed PE anti-human MRGPRX2 antibody staining on both non-transfected and stable H_MRGX2-transfected RBL-2H3 cells. The experimental results showed no morphological or growth state differences between the two cell types under bright-field microscopy (Figure 1b,d). Under the laser confocal microscope with Detection channel PE wavelength: Ex561 nm/Em560–610 nm, stable transfected RBL-2H3 cells displayed bright red fluorescence, while the non-transfected RBL-2H3 cells did not show any fluorescence under the same conditions (Figure 1a,c). Flow cytometry was used to quantitatively analyze fluorescence intensity, revealing that the stable transfected RBL-2H3 cells exhibited significantly stronger fluorescence intensity in the PE channel compared to the Mock-RBL-2H3 cells (*p* < 0.05) (Figure 1e,f). After 1 h of incubation with 20 μM of substance P, histamine levels were significantly increased in the H-MRGX2 RBL-2H3 cells (*p* < 0.05), whereas histamine levels were elevated but not significantly in the Mock-RBL-2H3 cells (*p* > 0.05) (Figure 1g).

### 3.3. Effects of Candidate Compounds on H_MRGX2 RBL-2H3 Cell Viability

**Figure 2:**
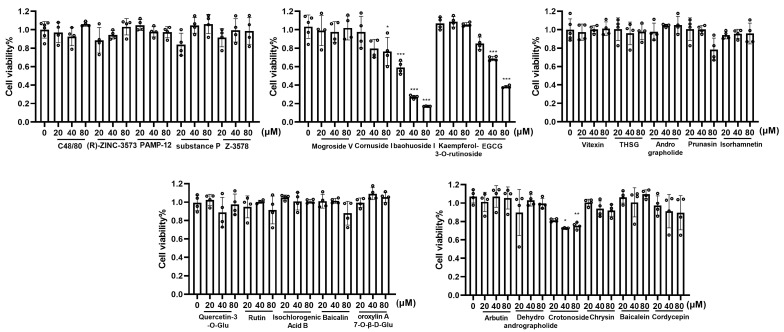
Effects of candidate compounds on cell viability (*n* = 4 in each group). Data are presented as means ± SD from three independent experiments. Compared with the control group, * *p* < 0.05,** *p* < 0.01, *** *p* < 0.001.

The CCK-8 assay revealed that H_MRGX2 RBL-2H3 cell viability was significantly inhibited after 1 h of intervention with Cornuside I (80 μM), Baohuoside I (20–80 μM), EGCG (40–80 μM), and Crotonoside (40–80 μM). Conversely, treatment with the remaining compounds for 1 h did not significantly affect H_MRGX2 RBL-2H3 cell viability compared to the untreated control group (Figure 2). Consequently, all subsequent cellular experiments utilized compound concentrations below 40 μM.

### 3.4. Candidate Compounds Triggered Intracellular Calcium Influx

**Figure 3:**
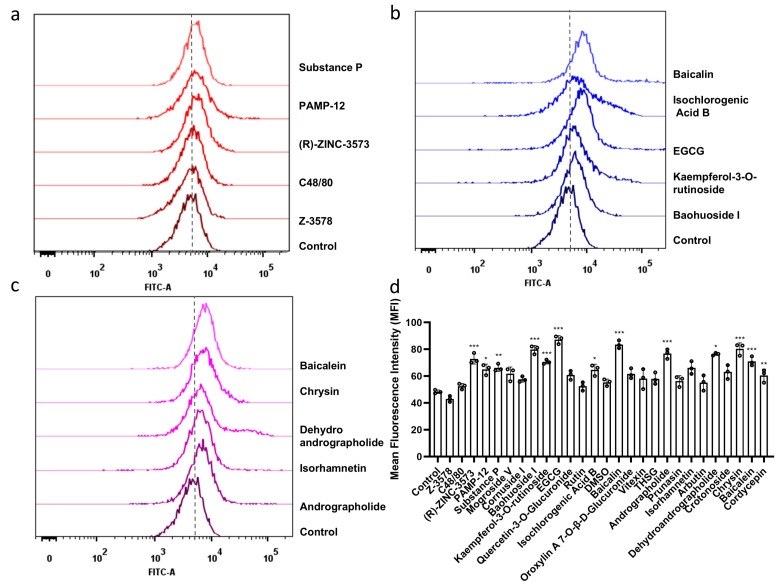
Intracellular Ca^2+^ was detected by flow cytometry, with fluorescence intensity in each group presented via overlay plots (**a**–**c**) and analyzed statistically (**d**) (*n* = 3 in each group). Data are presented as means ± SD from three independent experiments. Compared with the control group, * *p* < 0.05, ** *p* < 0.01, *** *p* < 0.001.

The calcium ion fluorescence intensity in the drug-intervened groups exhibited varying degrees of change compared to the control group (Figure 3). Among these, a significant increase in calcium ion fluorescence intensity was observed for (R)-ZINC-3573, substance P, PAMP-12, Baohuoside I, Kaempferol-3-O-rutinoside, EGCG, Isochlorogenic Acid B, Baicalin, Andrographolide, Isorhamnetin, Dehydroandrographolide, Chrysin, and Baicalein (*p* < 0.05).

### 3.5. Candidate Compounds Also Triggered Increased Tryptase and β-Hexosaminidase Levels in Mast Cells

**Figure 4:**
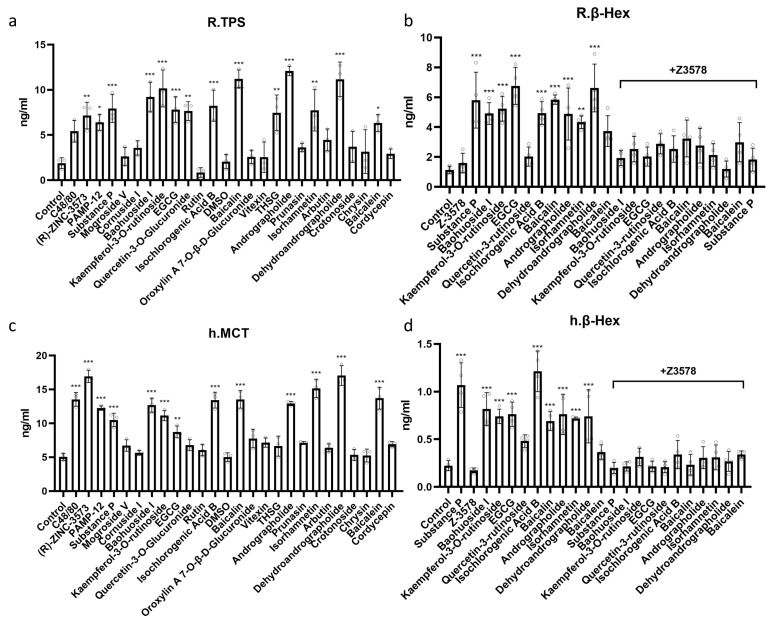
Quantification of mast cell degranulation compounds. Effects of candidate compounds on the secretion of tryptase (**a**) (*n* = 3) and β-hexosaminidase (**b**) (*n* = 4) from H_MRGX2 RBL-2H3 cells. Effects of candidate compounds on the secretion of tryptase (**c**) (*n* = 3) and β-hexosaminidase (**d**) (*n* = 4) from HMC-1 cells. Data are presented as means ± SD from three independent experiments. Compared with the control group, * *p* < 0.05, ** *p* < 0.01, *** *p* < 0.001.

Compared to the control group, all compounds induced varying degrees of elevation in tryptase and β-hexosaminidase in the H_MRGX2 RBL-2H3 and HMC-1 cells after 1 h of intervention (Figure 4). Notably, Baohuoside I, Kaempferol-3-O-rutinoside, EGCG, Isochlorogenic Acid B, Baicalin, Andrographolide, Isorhamnetin, and Dehydroandrographolide significantly increased both tryptase and β-hexosaminidase, with the exception of the positive control group. The induced degranulation could be inhibited by the MRGX2 inhibitor Z3578 (20 μM).

### 3.6. Candidate Compounds Induce Degranulation and Morphological Changes in H_MRGX2 RBL-2H3 Cells

**Figure 5:**
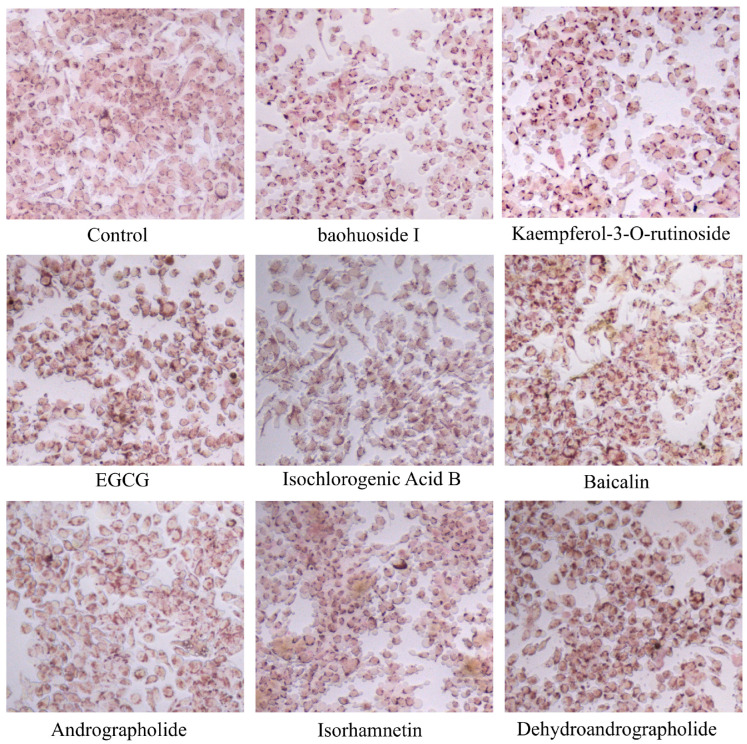
Microscopic Morphology of H_MRGX2 RBL-2H3 Cells Stained with Neutral Red (200×).

Neutral red staining revealed that control group were spindle-shaped, lightly stained, with intact membranes and dense morphology, showing no degranulation. Conversely, cells in the positive natural compound groups (treated with 20 μM of Kaempferol-3-O-rutinoside, EGCG, Baicalin, Andrographolide, Isorhamnetin, and Dehydroandrographolide; Baohuoside I at 10 μM) exhibited dark, brownish-red, round or oval shapes, along with microscopic signs of swelling, deformation, incomplete cell membranes, and granule exocytosis, evidenced by scattered red-stained extracellular granules. Only the control group and the neutral red-positive groups are shown in the figure (Figure 5), as no degranulation was observed with neutral red staining in the other compound-treated groups.

### 3.7. Protein-Small Molecule Interaction Poses and Binding Sites

**Figure 6:**
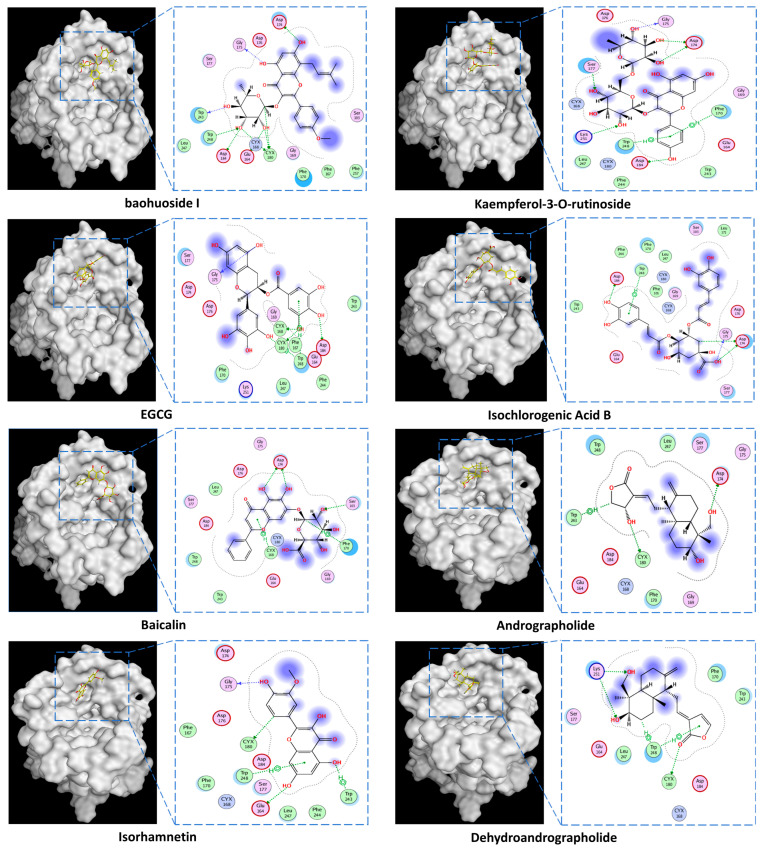
MRGPRX2 protein surface structure, the binding pocket location for natural compounds, and their interaction patterns in the optimal conformations.

The protein crystal structure of the target was obtained from the PDB database. Chemical structures of Baohuoside I, Kaempferol-3-O-rutinoside, EGCG, Isochlorogenic Acid B, Baicalin, Andrographolide, Isorhamnetin, and Dehydroandrographolide were drawn using ChemDraw software 23.0. Molecular docking was performed using predefined binding pocket locations and grid box sizes. The docking results were visualized as interaction poses (3D) and binding sites (2D), as shown in Figure 6.

## 4. Discussion

In vitro assays for evaluating pseudoallergic reactions offer advantages over in vivo methods, including rapid and sensitive detection, and suitability for high-throughput drug screening. The mast cell degranulation model is the most widely used in vitro approach for assessing pseudoallergic reactions [23]. These models typically utilize stable in vitro cell lines related to mast cells or basophils. Commonly used mast cell lines include RBL-2H3, mouse mastocytoma cells (P815) [24], and human mast cell lines such as human peripheral basophilic leukemia cells (Ku812), human mast cell LAD2, and HMC-1 [25,26]. Both LAD2 and HMC-1 cells express the MRGPRX2 receptor [27,28]. While LAD2 cells have a high granule content, they exhibit a long growth cycle [29], and these cell lines are often prone to contamination and difficult to obtain [30]. Rat basophilic leukemia RBL-2H3 cells are adherent, characterized by rapid growth, and possess readily observable basophilic granules. Mrgprb2 and Mrgprb3 are the murine and rat orthologs of MRGPRX2, respectively [31]. The murine ortholog Mrgprb2 shares only approximately 53% sequence homology with human MRGPRX2, and significant differences exist in their sensitivity to agonists and responses to inhibitors [32]. To facilitate direct observation of degranulation morphology, we humanized RBL-2H3 cells. Therefore, this study employed parallel experiments using HMC-1 cells and humanized MRGPRX2-expressing RBL-2H3 cells.

**Figure 7:**
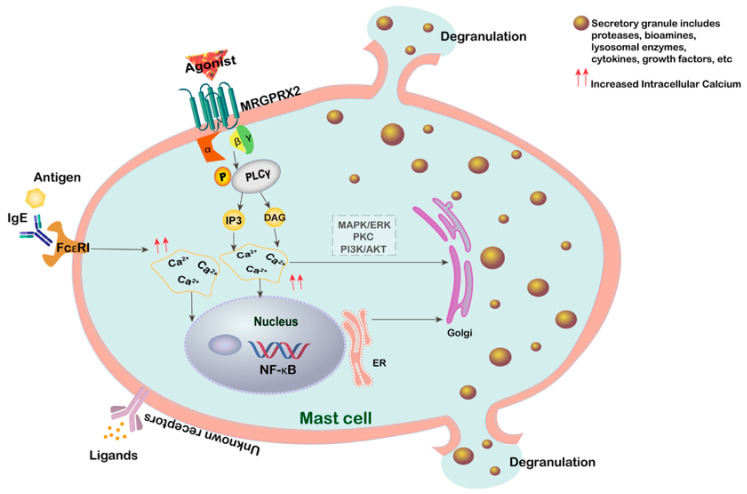
Mechanism diagram of mast cell degranulation. Mast cell degranulation is triggered by MRGPRX2 ligand activation (anaphylactoid reaction), antigen-IgE antibody activation of FcεRI (allergic reaction), and other unknown ligand–receptor interactions.

Mast cell degranulation is a hallmark of mast cell activation [33]. In IgE-independent hypersensitivity reactions, mast cell degranulation is triggered through MRGPRX2, rather than FcεRI [34,35]. The compounds released during mast cell degranulation include a variety of lysosomal enzymes, proteases, biogenic amines, proteoglycans, cytokines, and growth factors [36]. Histamine, tryptase [37], and β-hexosaminidase [38] are commonly used markers for assessing mast cell degranulation (Figure 7). However, histamine has a short half-life, and its detection sensitivity requires further improvement [39]. In this study, we used tryptase and β-hexosaminidase as indicators to assess the degranulation of H_MRGX2 RBL-2H3 and HMC-1 cells following compound treatment. Upon activation, the MRGPRX2 receptor couples to G proteins, initiating downstream signaling pathways. Specifically, MRGPRX2 can activate phospholipase C (PLC), leading to the production of second messengers such as inositol trisphosphate (IP3) and diacylglycerol (DAG). These molecules further activate protein kinase C (PKC) and other signaling molecules, ultimately resulting in intracellular calcium influx and the regulation of gene expression [40,41]. The downstream signaling cascades are complex. The activated MAPK/ERK signaling pathway plays a significant role in modulating and amplifying the response, for example, serving as a specific regulatory mechanism for IL-4 production in mast cells [42], and promoting late-phase inflammation [43]. The PI3K pathway is another crucial intracellular cascade in anaphylactoid/allergic reactions, linking cell survival and immune regulation, primarily responsible for maintaining calcium signaling [44], and closely associated with pathogenic mechanisms [45]. Additionally, the JAK/STAT and NF-κB pathways are also involved in regulating cytokine production [46]. Overall, throughout the entire process of allergic and anaphylactoid reactions, these complex signaling pathways exhibit crosstalk and synergistic activation [47], necessitating further in-depth investigation.

Analyzing the combined results from molecular docking, neutral red staining, calcium ion detection, and ELISA experiments, we found that 14 out of 22 natural compounds identified through molecular docking either exhibited weak degranulation effects or potentially acted as MRGPRX2 receptor inhibitors, excluding the positive control. The remaining eight natural compounds, including Baohuoside I, Kaempferol-3-O-rutinoside, EGCG, Isochlorogenic Acid B, Baicalin, Andrographolide, Isorhamnetin, and Dehydroandrographolide, significantly activated the MRGPRX2 receptor, triggering mast cell degranulation. Specifically, Baohuoside I, a flavonoid compound derived from Epimedium koreanum [48], is a component used for the content determination of Epimedium in the Chinese Pharmacopoeia. Kaempferol-3-O-rutinoside is found in Carthamus tinctorius [49] and Tetrastigma hemsleyanum, and caution should be exercised regarding appropriate dosage when using injections containing Carthamus tinctorius (e.g., Honghua injection, Xuebijing injection) [50]. EGCG is primarily extracted from green tea and is also present in Ginkgo Semen, Eriobotryae Folium, and Phyllanthi Fructus [51]. Isochlorogenic Acid B, a polyphenol compound extracted from Lonicera japonica, is a component of Shuanghuanglian injection and Reduning injection [52,53]. Baicalin is a component used for identification and quantitative analysis of Scutellariae Radix in the Chinese Pharmacopoeia, and related injections such as Yinhuang injection, Qingre Jiedu injection, and Yinzhihuang injection contain this component [54]. Moreover, previous studies have shown that Baicalin is a key contributor to ARs observed with Shuanghuanglian injection in clinical practice [55]. Andrographolide and dehydroandrographolide are quantitative markers for Andrographis paniculata in the Chinese Pharmacopoeia and are present in the Andrographis paniculata injection, Chuanhuning injection, and Xiyanping injection [56]. Lin et al. identified dehydroandrographolide as a potential allergenic component of Andrographis paniculata injection [57]. Isorhamnetin is one of the most important active compounds in Hippophae rhamnoides fruit and Ginkgo biloba leaves [58]. It is present in Ginkgo biloba extract injections and Ginkgo biloba Damo Injection.

Given these findings, special attention should be paid to potential pseudoallergic reactions in patients when using TCM preparations containing the aforementioned compounds. Considering individual variability in constitution and receptor expression levels, individuals with allergic predispositions may be more sensitive to these sensitizing substances. Therefore, the specific clinically controlled doses of these compounds that potentially induce pseudoallergic reactions, as identified in this study, require evaluation with more clinical data. In conclusion, this study successfully established an MRGPRX2-mediated mast cell degranulation model, providing a solid foundation for further investigation of the mechanisms underlying pseudoallergic reactions caused by TCM and TCMI. In the future, we will combine more clinical data to thoroughly evaluate the specific clinically controlled doses of the natural compounds identified in this study that potentially induce pseudoallergic reactions, with the aim of providing more informative guidance for the quality control, safety re-evaluation, and risk assessment of TCM and TCMIs.

## 5. Conclusions

In this study, we discovered that specific natural compounds can induce mast cell degranulation and trigger ARs by activating the MRGPRX2 receptor. This finding provides crucial molecular insights into the rapid-onset ARs observed during the clinical application of certain natural compounds, particularly TCMI ingredients. It highlights potential risks, contributes to enhanced medication safety, and lays the groundwork for the quality control and safety re-evaluation of TCM.

This study has several limitations. For example, we mainly focused on establishing an anaphylactoid cell model and evaluating the in vitro effects of natural compound induced ARs through computer-aided screening. Most of the experiments were validated on the H_MRGX2 RBL-2H3 model, which may differ from primary mast cells, and thus require cautious extrapolation. Furthermore, there is a lack of investigation into other receptors and signaling pathways. Future research can focus on further exploring the allergic or pseudo-allergic reactions of these natural products in vivo, as well as the molecular signals involved. Additionally, the exploratory methods employed in this study could offer valuable insights for applications in other related fields.

## Figures and Tables

**Table 1:** Ranked results of natural compounds exhibiting significant binding efficacy.

Pubchem Cid	Name	Mol	S	Rmsd_Refine	E_Conf	E_Place	E_Score	E_Refine
24721270	Mogroside V	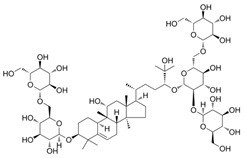	−11.2304	3.1565	559.814	−104.5097	−8.3637	−78.0384
11228694	Cornuside I	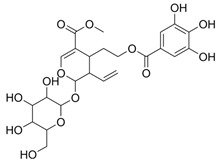	−7.4277	3.1595	98.4467	−83.0819	−9.6318	−41.4245
5488822	Baohuoside I	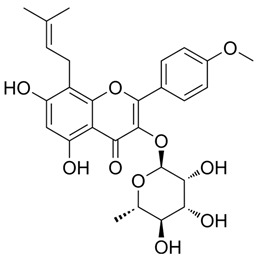	−7.1238	1.2427	79.4959	−99.0347	−10.2188	−39.2266
5318767	Kaempferol-3-O-rutinoside	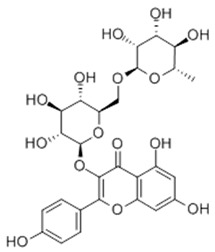	−7.0874	2.8019	134.5548	−67.8698	−9.2520	−48.6317
65064	EGCG	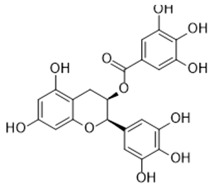	−6.7253	2.4423	−29.3672	−75.5645	−11.3901	−36.9659
5274585	Quercetin-3-O-Glucuronide	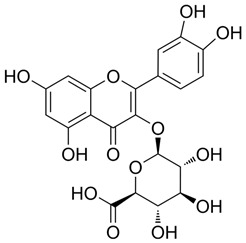	−6.5532	1.8696	79.1429	−70.3006	−10.2403	−40.0644
5280805	Rutin	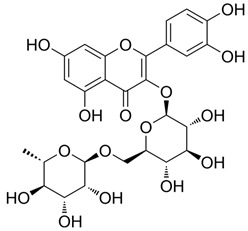	−6.5235	2.0524	148.7302	−67.8863	−11.5230	−38.5485
5281780	Isochlorogenic Acid B	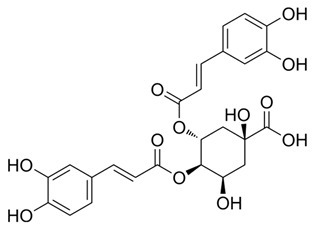	−6.4882	1.7029	−38.7087	−43.3100	−9.5616	−32.1427
245005	Aconitine	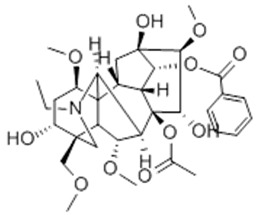	−6.2575	3.6729	278.6369	−46.8533	−8.0642	−34.1713
64982	Baicalin	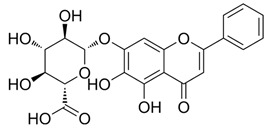	−6.0956	1.9665	71.7165	−85.7982	−10.2547	−31.9911
14655552	Oroxylin A 7-O-β-D-Glucuronide	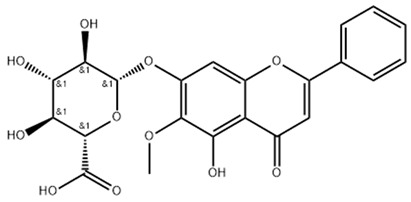	−5.8501	1.9144	85.5989	−82.7878	−9.1929	−31.7040
5280441	Vitexin	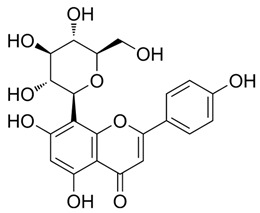	−5.8363	1.1508	46.8506	−87.0438	−11.3955	−31.6867
5321884	THSG	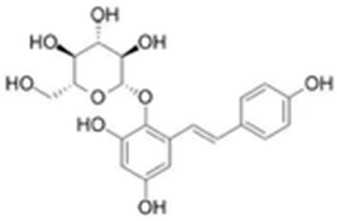	−5.5554	1.5307	56.4074	−72.9719	−10.3949	−25.6776
5318517	Andrographolide	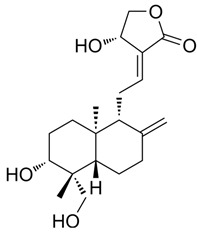	−5.4928	1.873	75.2459	−66.9751	−8.5375	−22.1143
119033	Prunasin	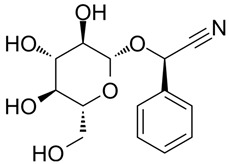	−5.4698	2.9089	105.2935	−63.6619	−8.2991	−26.5614
5281654	Isorhamnetin	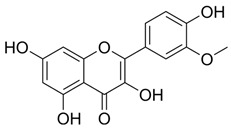	−5.4472	1.1922	20.4338	−66.4913	−10.4481	−29.8052
440936	Arbutin	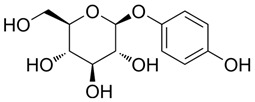	−5.4392	1.1603	68.5812	−64.0948	−8.9051	−28.5810
78577438	Dehydroandrographolide	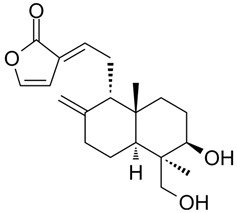	−5.3330	1.6409	107.6252	−64.4499	−9.5483	−23.5010
65085	Crotonoside	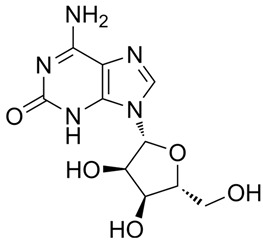	−5.1651	1.2359	−46.4225	−61.3834	−9.1330	−24.2
5281607	Chrysin	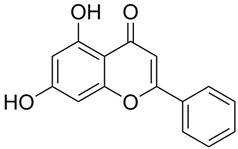	−5.0591	2.2533	−12.9074	−51.8085	−9.8103	−23.9324
5281605	Baicalein	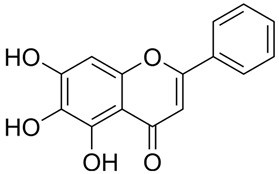	−5.0570	1.7595	1.3269	−61.1301	−10.8902	−23.7111
6303	Cordycepin	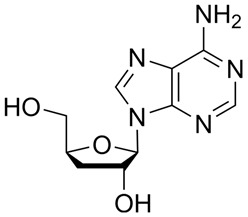	−5.0094	1.2783	15.0973	−61.5090	−9.5804	−22.7595

## Data Availability

The original contributions presented in this study are included in the article. Further inquiries can be directed to the corresponding authors.

## Abbreviations

The following abbreviations are used in this manuscript:

ARs	Anaphylactoid reactions
C48/80	Compound 48/80
DAG	Diacylglycerol
EGCG	Epigallocatechin gallate
IgE	Immunoglobulin E
IP3	Inositol trisphosphate
MCs	Mast cells
MOE	Molecular Operating Environment
MRGPRX2	Mas-related G protein-coupled receptor X2
PKC	Protein kinase C
PLC	Phospholipase C
TCMIs	Traditional Chinese Medicine Injections
TCM	Traditional Chinese Medicine
THSG	2,3,5,4-tetrahydroxyl diphenylethylene-2-o-glucoside
